# Perirenal Extravasation After Retrograde Intrarenal Surgery for Renal Stones: A Prospective Study

**DOI:** 10.7759/cureus.21283

**Published:** 2022-01-16

**Authors:** Anil Shrestha, Binod B Gharti, Baikuntha Adhikari

**Affiliations:** 1 Department of Urology, Bir Hospital, National Academy of Medical Sciences, Kathmandu, NPL

**Keywords:** systemic inflammatory response syndrome, retrograde intrarenal surgery, intrarenal pressure, laser, extravasation

## Abstract

Background

The incidence and consequences of the perirenal extravasation of the irrigation fluid during retrograde intrarenal surgery (RIRS) are not fully elucidated. The objective of this study was to assess the incidence, risk factors, and complications of perirenal extravasation of irrigation fluid during RIRS.

Methods

This prospective observational study was conducted in the Department of Urology, Bir Hospital, Kathmandu, Nepal, from January 2020 to March 2021. Patients undergoing RIRS for renal stones less than 2 cm in diameter were included in the study. Irrigation during the procedure was performed using isotonic normal saline under gravity at 50 cm from the symphysis pubis of patients with intermittent manual compression if required. Lithotripsy was performed with 120-Watt Ho:YAG laser using 200-micron laser fiber. Retrograde pyelogram was performed after the completion of lithotripsy to document the presence or absence of extravasation on fluoroscopic images. A double-J stent was placed at the end in all patients. Patients were observed for systemic inflammatory response syndrome (SIRS) features. Postoperative abdominal ultrasonography was performed on the first postoperative day to assess the perirenal collection together with complete blood count. The visual analogue scale (VAS) was used to assess pain in postoperative period. The preoperative patient’s and stone characteristics, hydronephrosis, intraoperative characteristics, and postoperative findings were analyzed.

Results

A total of 71 patients who underwent RIRS during the study period were analyzed. The mean (SD) stone size was 13.19 (3.12) mm. Intraoperative perirenal extravasation of contrast was noted in eight (11.26%) patients; however, no patient had ipsilateral perirenal collection on ultrasonography obtained on the first postoperative day. No significant differences were observed among patients with or without perirenal extravasation in terms of mean stone size, laser settings, operative duration, and lasing duration. Use of ureteral access sheath (UAS) was associated with lower incidence of extravasation; however, it was not significant. SIRS was documented in eight patients overall, with none of the patients with extravasation having features of SIRS. Patients with extravasation experienced more postoperative flank pain (p<0.05).

Conclusion

Perirenal extravasation was common during RIRS and was associated with higher postoperative pain scores. Stone size, use of UAS, laser settings, operative duration, and lasing duration were not associated with an increased risk of extravasation. Extravasation was not associated with increased postoperative complications.

## Introduction

Retrograde intrarenal surgery (RIRS) is increasingly being a preferred procedure for renal stones by patients and surgeon alike owing to its low complication rate and minimal invasive nature [1]. With surgery being performed in the closed system, increased intrarenal pressure is expected with irrigation and instrumentation [2]. Auxiliary devices such as ureteral access sheath (UAS) have been shown to decrease intrarenal pressure during RIRS by allowing continuous irrigation outflow. However, increased intrarenal pressure 20-30 times above normal has been documented during flexible ureteroscopy [3]. In addition to significant pyelovenous and pyelolymphatic absorption increasing the risk of infectious complications, sustained increased intrarenal pressure during RIRS can lead to forniceal rupture with attendant increased risk of subcapsular hematoma, perirenal hemorrhage, or collection [3-5].

Although perirenal extravasation of the irrigation fluid is a recognized complication, its exact incidence and consequences are sparsely reported in the existing literature. The objective of this study was to assess the incidence, risk factors, and complications or sequelae of perirenal extravasation of irrigation fluid during RIRS.

## Materials and methods

This prospective observational study was conducted in the Department of Urology, Bir Hospital, National Academy of Medical Sciences, Kathmandu, Nepal, from January 2020 to March 2021. Ethical approval was obtained from the Institutional Review Board of National Academy of Medical Sciences, and written informed consent was obtained from all patients.

Patients older than 14 years with renal stones less than 2 cm in diameter undergoing RIRS were included in the study. Patients with no preoperative CT scan, bilateral procedures, obstructed system, ureteral stricture, ureteric stones, renal anomaly, simultaneous endopyelotomy, simultaneous infundibulotomy, and calyceal diverticular stone were excluded. Patient demographics including age, sex, BMI, relevant medical history, and CT KUB/IVU (kidneys, ureters, and bladder/intravenous urogram) were obtained to assess hydronephrosis, the stone location, density, and Hounsfield unit. Sterile urine culture was obtained before surgery.

Surgical procedure

All the surgeries were performed by a single consultant urologist (A.S.). Prophylactic antibiotic (ceftriaxone 1 gm) was administered prior the induction of general anesthesia, and the patient was placed in the lithotomy position. Irrigation during surgery was performed under gravity using 1 L of normal saline, with the lower margin of the irrigation bag maintained at a height of 50 cm from the level of the symphysis pubis of the patient with an intermittent manual pump with Pathfinder or TraxerFlow™ (Rocamed, Monaco). Cystoscope was used to place hydrophlic guidewire (0.035”, Terumo, Tokyo, Japan) upto the ipsilateral pelvicalyceal system under fluoroscopic guidance. The ureteric access sheath (UAS) (Cook 9.5/11.5 UAS for Flex X2S, Cook Medical, Bloomington, IN, USA, and Olympus 10/12 UAS for URF V3, Olympus, Tokyo, Japan) was routinely used if negotiable, and sheathless procedure was performed in cases where the UAS was not negotiable. Inspection of all calyces was performed, and stone considered in unfavorable location for laser lithotripsy in situ by the operating surgeon was relocated before lithotripsy using various zero tip nitinol baskets (N-circle nitinol basket extractor, 2.2-Fr 115-cm basket, Cook Medical). Laser lithotripsy was performed using Ho:YAG laser (120H Lumenis) using 200-micron laser fiber. Laser lithotripsy was performed employing the following settings:

(1) high power setting: Dusting - 0.2-0.5J and 40-70 Hz; fragmentation - 1J and 10 Hz; pop dusting - 0.5J and 50 Hz

(2) low power setting: Dusting - 0.5 -0.8J and 10-15 Hz; fragmentation - 1J and 10 Hz, popcorning - 1-1.2J and 15-20 Hz

Lithotripsy was performed till the fragments were easily floated or dislodged with gentle irrigation. At the completion of lithotripsy, retrograde pyelogram (RPG) was performed using 1:1diluted contrast diatrizoate meglumine and diatrizoate sodium (76%), and 5 mL of solution was injected slowly each time with fluoroscopy to the maximum volume of 10 mL over 10-15 seconds. Presence or absence of extravasation of contrast was documented. A double-J stent was inserted in all cases at the end.

Intravenous analgesics (Paracetamol six hourly) and oral alpha blocker (alfuzosin 10 mg at bed time) were administered in the postoperative period. Patients were given injection ketorolac and/or tramadol in case of inadequate pain relief. Postoperatively, patients’ vital parameters were monitored (pulse rate, blood pressure, temperature, respiratory rate) hourly, and severity of the flank pain was assessed using visual analogue scale (VAS) on the first postoperative day three hours after the last dose of the parenteral analgesia. On the first postoperative day, complete blood count and ultrasound of the abdomen (to assess for ipsilateral perirenal collection) were performed. Patients were usually discharged on the first postoperative day. The double-J stent was removed after 14 days, and patients were followed up till four weeks.

The operative duration was considered from the time of placement of cystoscope for guidewire placement till the placement of the double-J stent at the end of procedure. The total lasing energy utilized and lasing duration were recorded. Pain severity was assessed using VAS and categorized as 0 for no pain, 1-2 for mild pain, 3-5 for moderate pain, 6-7 for severe pain, 8-9 for very severe pain, and 10 for the worst pain possible. Systemic inflammatory response syndrome (SIRS) was recorded in the immediate postoperative period. Presence of equal to or more than two components of SIRS was considered significant [6]. Complication during this period was graded using the modified Clavien-Dindo classification system [7].

Data analysis was performed using the IBM Statistical Package for Social Sciences (SPSS Version 20 for Windows, IBM Corp., Armonk, NY, USA). Baseline characteristics were compared using the chi-square test/Fisher’s exact test for categorical variables and t-test for continuous variables. A p-value of less than 0.05 was considered statistically significant.

## Results

A total of 71 patients were included in the final analysis after exclusion of seven patients. Patients with simultaneous infundibulotomy (n=3), diverticular stone (n=3), and pelvic kidney (n=1) were excluded from the final analysis.

Majority of the participants were male and the mean (SD) of the participants was 40.58 (11.36) years (Table 1). The mean size of stone was 13.19 ± 3.12 mm, with most of the stones located in the lower pole (n=23, 32.3 %) and mid pole (n=20, 28.1%). Preoperative double-J stent placement was not performed in the majority of the patients (94.4%) (Table 1).

**Table 1:** Basic characteristics of patients Data are presented as mean ± SD and as number (percentage)

Variables	Mean ± SD
Age (years)	40.58 ± 11.36
Sex	
Male	42 (59.2%)
Female	29 (40.8%)
BMI (kg/m^2^)	22.83 ± 1.3
Stone size (mm)	13.19 ± 3.12
Mean stone density (HU)	944 ± 112
Stone location	
Pelvis	14 (19.7%)
Upper pole	14 (19.7%)
Mid pole	20 (28.1%)
Lower pole	23 (32.3%)
Preoperative double-J stent	
Yes	4 (5.6%)
No	67 (94.4%)

The perirenal extravasation was present in eight patients (11.2%) during intraoperative RPG. The mean stone size was similar between those with or without extravasation (p=0.513). The UAS was used in 60 (84.5%) patients. Extravasation was present in six patients and two patients with or without UAS use, respectively. Lithotripsy was performed in situ in 53 (74.6 %), and among those with relocation (n=18), majority were relocated to the upper pole calyx (n=16 [22.5%]) followed by the middle pole calyx (n=2 [2.8%]) (Table 2). There was no difference in extravasation in relation to relocation (p=0.409). The mean operative duration, lasing duration, and laser power settings were similar between those with or without extravasations intraoperatively. The intraoperative perirenal leak was identified in 18.1% (two out of 11) and 10% (six out of 60) patients with and without the use of UAS, respectively; however, it was not statistically significant (p=0.601) (Table 2).

**Table 2:** Relation of perirenal leak with intra- and postoperative parameters Data are presented as mean ± SD and as number (percentage) POD, postoperative day; RPG, retrograde pyelogram; SIRS, systemic inflammatory response syndrome; UAS, ureteral access sheath; VAS, visual analogue scale

		RPG leak		p-Value
	Overall	Presence (n=8)	Absence (n=63)	
Operative duration (minutes)	28.52 ± 6.72	30.00 ± 6.547	28.33 ± 6.780	0.513
Lasing time (seconds)	519. 94 ± 27.33	517.00 ± 25.67	520.32 ± 27.7	0.74
Stone size (mm)	13.19 ± 3.12	12.61 ± 1.95	13.27 ± 3.24	0.57
UAS use				0.601
No (n)	11 (15.5 %)	2	9	
Yes (n)	60 (84.5%)	6	54	
SIRS				0.786
Presence	2	0	2	
Absence	69	8	61	
Power setting				0.629
Low power	35 (49.3%)	4	31	
High power	36 (50.7%)	4	32	
Relocation				0.409
Done	18 (23.4 %)	3	15	
Not done	53 (74.6%)	5	48	
VAS grade				0.024
Mild pain	59 (83.1%)	4	55	
Moderate pain	12 (16.9%)	4	8	
Perirenal collection on the first POD	None	None	None	

Mild postoperative pain was present in 59 (83.1%) patients and moderate postoperative pain was present in 12 (16.9%) patients, and the pain intensity assessed by VAS was significantly higher in patients with extravasation (p=0.024). Although eight patients had RPG detected extravasation, none of the patients had perirenal collection or parenchymal or subcapsular hematoma in ultrasonography obtained on the first postoperative day. Overall, SIRS was documented in two patients and RPG leak was not associated with the incidence of SIRS (p=0.786). There were no major complications as three patients developed postoperative complications, with one patient having grade 1 and two patients having grade 2 complications according to the Clavien classification (Table 2).

## Discussion

Increased intrarenal pressure is expected during RIRS as it is performed in a closed pelvicalyceal system. Sustained increased or intermittent increased intrarenal pressure during manual irrigation can lead to perirenal extravasation of irrigation fluid due to forniceal rupture [4]. In this study, perirenal extravasation was documented in 11.2% and was associated with higher pain scores than those without it; however, it was not associated with increased complication rate.

The normal intrarenal pressure is identified to be less than 10 mmHg (13 cmH_2_O) while the threshold for pyelovenous and pyelosinous absorption has been shown to be in the order of 30-45 mmHg (40-60 cmH_2_O), and sustained pressure above that limit may lead to forniceal rupture resulting in perirenal extravasation [2,4]. The incidence of subcapsular hematoma following semirigid ureteroscopy for ureteric calculus was 0.15-0.36% in retrospective analysis; however, there is a paucity of published literature on RIRS [8,9]. In our study, eight (11.2%) patients had perirenal extravasation during intraoperative pyelogram; however, none of them had perirenal collection or hematoma collection on the ipsilateral side in ultrasonography performed on the first postoperative day.

Studies had documented an increased risk of infectious complications with increased intrarenal pressure. Since perirenal extravasation is also a consequence of increased intrarenal pressure, it may be associated with increased complications. In our study, overall febrile illness occurred in three (4.2%) patients, and none of the patients developed sepsis. Similar observations were noted by Berardinelli et al. in their study on infective complications in RIRS [10]. In our study, no febrile illness or infective complications were noted in those with perirenal extravasation, and SIRS was present in two of 71 patients. In fact, all the patients who developed infective complication/SIRS had no perirenal extravasation. Preoperative sterile urine culture and only intermittent gentle manual irrigation with predominant irrigation under gravity may have led to the reduced incidence of SIRS or infective complications in our study.

There was no significant difference observed in the patients with or without perirenal leak in relation to the mean stone size, average lasing time, and laser power setting (high versus low). High power laser settings can lead to increased temperature on the system and increased chances of injury to mucosa with resultant increased risk of extravasation. However, no relation with laser settings was observed in our study. The larger stone size would require longer duration for lithotripsy, and the increased number of fragments would more likely to impair vision, thus necessitating manual irrigation as well as longer operative duration and hence increasing the risk of extravasation; however, no such relation was observed in our study. Regular use of UAS and relocation of stone in unfavorable location before lithotripsy would have led to the results in our study.

The use of UAS has been shown to reduce intrarenal pressure [11]. In our study, a slightly higher rate of perirenal leak was observed in cases where UAS was not used, although it was not significant.

In our study, most patients (83%) had mild pain while the remaining 17% had moderate pain in the postoperative period assessed using VAS, and a significantly higher number of patients with extravasation experienced moderate pain. It could be explained by the increased distension of the renal capsule. The available literature remains unclear regarding the associated factors with pain after flexible ureteroscopy. However, the larger stone burden, prolonged operative duration, and duration of the UAS in situ were associated with significantly higher pain scores after laser lithotripsy with semirigid ureteroscopy [12].

The effect of a ureteral J-stent on postoperative pain inserted at the end of the operation is still debatable [12]. While Torricelli et al. found reduced pain with the placement of the double-J stent in the postoperative period, Byrne et al. reported increased discomfort in those with stent placement [13,14]. In our study, a double-J stent was inserted in all patients.

Simultaneous intrarenal pressure measurement was not performed in our study to correlate the relation with perirenal extravasation. Small sample size limits the generalizability of the results.

## Conclusions

Minor perirenal extravasation of irrigation fluid was not uncommon during RIRS for renal stones. The presence of extravasation is usually associated with significantly higher flank pain in the postoperative period. The extravasation detected intraoperatively resolved within the first postoperative day without major sequelae.

Measures to reduce intrarenal pressure such as use of UAS and judicious use of manual irrigation intraoperatively and postoperative use of a double-J stent may help reduce the risk of perirenal extravasation.
